# Role of early growth response 1 in liver metabolism and liver cancer

**DOI:** 10.20517/2394-5079.2017.36

**Published:** 2017-11-20

**Authors:** Nancy Magee, Yuxia Zhang

**Affiliations:** Department of Pharmacology, Toxicology & Therapeutics, University of Kansas Medical Center, Kansas City, KS 66160, USA

**Keywords:** Early growth response 1, liver, fibrosis, injury, liver cancer

## Abstract

The liver is an essential organ for nutrient and drug metabolism - possessing the remarkable ability to sense environmental and metabolic stimuli and provide an optimally adaptive response. Early growth response 1 (Egr1), an immediate early transcriptional factor which acts as a coordinator of the complex response to stress, is induced during liver injury and controls the expression of a wide range of genes involved in metabolism, cell proliferation, and role of Egr1 in liver injury and repair, deficiency of Egr1 delays liver regeneration process. The known upstream regulators of Egr1 include, but are not limited to, growth factors (e.g. transforming growth factor β1, platelet-derived growth factor, epidermal growth factor, hepatocyte growth factor), nuclear receptors (e.g. hepatocyte nuclear factor 4α, small heterodimer partner, peroxisome proliferator-activated receptor-γ), and other transcription factors (e.g. Sp1, E2F transcription factor 1). Research efforts using various animal models such as fatty liver, liver injury, and liver fibrosis contribute greatly to the elucidation of Egr1 function in the liver. Hepatocellular carcinoma (HCC) represents the second leading cause of cancer mortality worldwide due to the heterogeneity and the late stage at which cancer is generally diagnosed. Recent studies highlight the involvement of Egr1 in HCC development. The purpose of this review is to summarize current studies pertaining to the role of Egr1 in liver metabolism and liver diseases including liver cancer.

## INTRODUCTION

**Figure 1:**
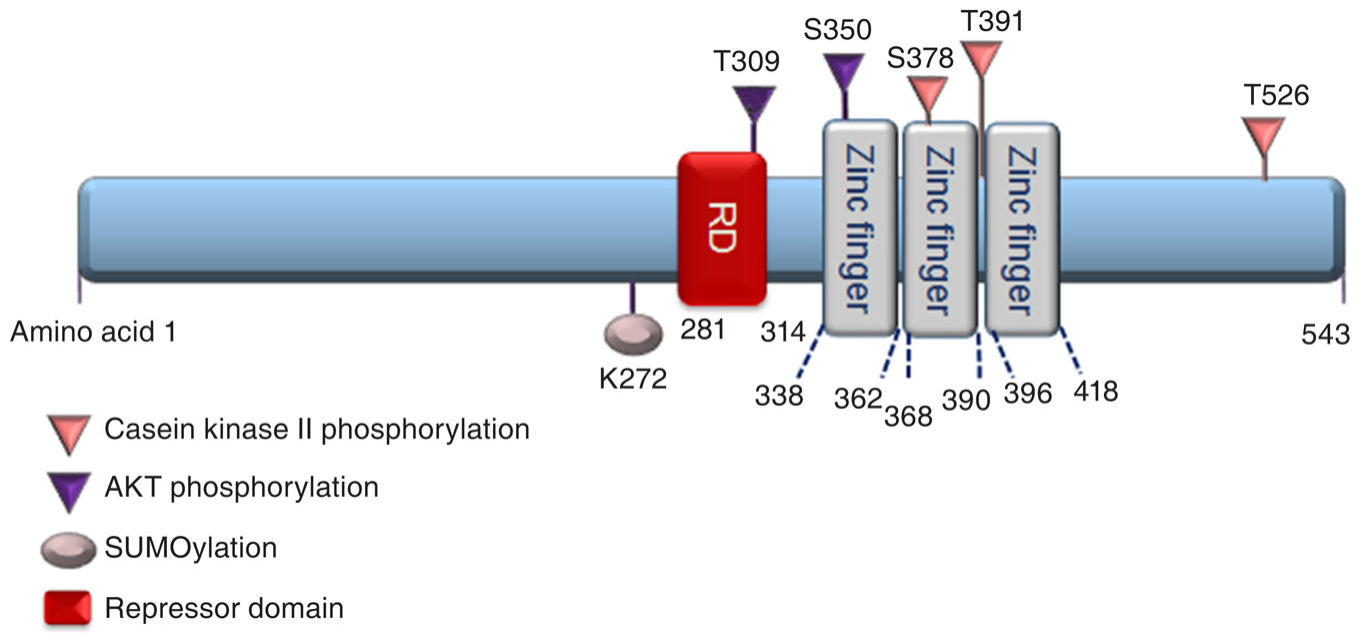
Schematic representation of EGR1 protein structure and post-translational modifications. EGR1 is a 543-amino acid (aa) protein consisting of three Cysteine 2-Histidine 2 (C_2_H_2_) zinc fingers DNA-binding domains, approximately 23 aa each. Zinc fingers 2 and 3 (amino acids 361–419) interact with amino acids 315–330 for EGR1 nuclear localization. The T309 and S350 sites are phosphorylated by protein kinase B (PKB, also known as AKT); whereas, S378, T391, and T526 sites are phosphorylated by casein kinase II. EGR1 protein can be SUMOylated by SUMO1 at K272. Transcriptional co-repressors NGFI-A binding protein 1and 2 (NAB1 and NAB2, respectively) inhibit Egr1 transcriptional activity by binding to the repressor domain (RD). EGR1: early growth response 1

Early growth response 1 (Egr1) is an immediate early, zinc finger transcription factor that was first identified based upon its induction by nerve growth factor (NGF) in rat PC12 cells, which is why it was initially known as nerve growth factor inducible protein A (NGFI-A)^[1]^. Egr1 is one of four family members that also include Egr2, Egr3, and Egr4^[2]^. Also known as *Krox24*, *zif268*, and *TIS8*, *Egr1* encodes a protein of 80–82 kDa that consists of three zinc finger DNA-binding motifs [Figure 1]. Thus, it is not elusive that zinc metal is crucial to the function of Egr1, such as nuclear localization^[3]^. Specifically, two of three zinc fingers interact with the nuclear localization sequence to promote Egr1 nuclear localization^[3]^. Depletion of the zinc metal reduces Egr1 promoter activity^[4]^. Transcriptional co-repressors NGFI-A binding protein 1 and 2 (NAB1 and NAB2, respectively) repress Egr1, Egr2, and Egr3 transcriptional activity by binding to the respective repressor domains upstream of the zinc finger motifs and could potentially co-regulate Egr1 target genes^[5–7]^.

Egr1 expression can be induced by growth factors, ionizing radiation^[8]^, and insulin signaling^[9]^. Upstream regulators of Egr1 include transforming growth factor β1 (TGF-β1)^[10]^, mitogen-activated kinase kinase-1, hepatocyte nuclear factor 4α, and E2F transcription factor 1 (E2F1); whereas small heterodimer partner and peroxisome proliferator-activated receptor-γ agonist are negative regulators of Egr1^[11–14]^. Egr1 recognizes a highly conserved G-C-rich consensus nucleotide sequences (GCGGGGGCG)^[15]^ and either activates or represses the transcription of genes in a zinc-dependent manner. The presence of this specific Egr1 response element on its target gene promoter could thus be a good indication of direct transcriptional regulation by Egr1.

The expression of Egr1 has been described in liver, heart, brain, spleen, skeletal muscle, kidney, ovary and prostate^[16]^. Accordingly, important roles of Egr1 has been implicated in various cell types and pertain to embryogenesis^[17]^, cell growth and differentiation^[18]^, neurogenesis^[19]^, adipogenesis^[20]^, apoptosis^[21]^, fibrogenesis^[22]^, and tumorigenesis^[23]^. *Egr1* is one of the predominantly expressed EGR family members in the liver and liver-derived cell lines^[24,25]^. Extensive research has been conducted in animal models to elucidate Egr1 function in various liver diseases. In this review article, we begin by discussing the role of Egr1 in liver metabolism, and then focus on Egr1 in pathological states of liver with a particular interest in hepatocellular carcinoma (HCC). An unbiased discussion of what additional studies are necessary to aid in developing possible therapeutic interventions is also included.

## EGR1 AND LIVER METABOLISM

Liver is a major site for synthesis, metabolism, storage and redistribution of glucose and lipids^[26]^. In the postprandial state, insulin is secreted from pancreatic beta cells in response to a high blood-sugar level. Circulating glucose is taken up by the hepatocyte via the glucose transporter type 2 - regulated by the serine/threonine kinase PI3K/AKT pathway in response to insulin signaling - and is phosphorylated to glucose-6-phosphate by liver glucokinase (Gck). Glucose-6-phosphate is either further processed for fuel via glycolysis, for nucleotide biosynthesis via pentose phosphate pathway or utilized for glycogen synthesis via glycogen synthase, depending on the systemic metabolic state. In addition, insulin further promotes *de novo* lipogenesis of fatty acids from acetyl-CoA or malonyl-CoA. In the fasting state, glucagon is secreted by the alpha cells of pancreas in response to a low blood-sugar level. Upon glucagon stimulation, the liver synthesizes glucose *de novo* as well as catabolizes glycogen to release glucose for other organs to use for energy. During this time, lipolysis in adipose tissues is increased and results in the production of free fatty acids, which is taken up by hepatocytes. Depending on the metabolic state, fatty acids are then either processed to triglycerides (TAGs) for storage or rapidly metabolized for the generation of ketone bodies that are, in part, oxidized by hepatic mitochondria. In the event of excess lipid accumulation in hepatocytes that exceeds 5% of liver weight, whether due to over nutrition or hyperglycemia, non-alcoholic fatty liver disease can develop. Thus, hepatic lipids can either derive from endogenous lipogenesis (*de novo* lipogenesis), which may account for up to 30% of TAGs in steatotic livers^[27]^, or derive from the active uptake of circulating fatty acids into the hepatocytes.

### Glucose and insulin regulate Egr1 expression

The contributions of glucose and insulin to Egr1 expression have been extensively studied in a variety of tissues and cell types. One earlier study showed that glucose rapidly and transiently induces Egr1 mRNA in SV40-transformed murine pancreatic beta-cell line MIN6 cells that is accompanied with an induction of insulin^[28]^. This study also demonstrated that the induction of Egr1 by glucose was unique to beta cells since glucose couldn’t induce Egr1 expression in NIH-3T3 fibroblasts or hepatocytes ^[28]^. The results raise a question whether glucose regulates Egr1 expression requires insulin signaling activation. Later, another study showed that in vascular endothelial cells, glucose and insulin independently regulated Egr1 expression and they had an additive effect to induce Egr1 in the co-treatment^[29]^. Specifically, glucose mediates its effects through activation of PKC while insulin acts through the extracellular signal-regulated kinase (ERK1/2) pathway^[29]^. Collectively, these studies suggest that glucose or insulin differentially regulates Egr1 expression in a cell-type dependent manner.

Insulin regulates Egr1 expression in hepatoma cells^[9]^ and in non-liver-derived cells overexpressed with insulin receptors^[30,31]^. Keeton *et al.*^[9]^ showed that in rat hepatoma H4IIE cells, insulin treatment rapidly and transiently induced Egr1 mRNA, reaching its maximum levels by 15 min, which was coordinately regulated by a regulatory network involving MAPK kinase (MEK)-ERK, p38 MAPK, and PI3-kinase (PI3K). In addition, the authors found that the activation of ERK1/2 was essential for the induction of Egr1 in response to insulin that could be further modulated by alterations in the activity of the p38 MAPK pathway^[9]^. By contrast, inhibition of the PI3K pathway augmented insulin’s effect on Egr1 expression, suggesting that some factor downstream of PI3K may partially inhibit induction of Egr1. Of particular interests, Egr1 has been implicated to mediate the regulation of insulin on genes in liver metabolism, including hepatic malic enzyme (ME)^[32,33]^ and apolipoprotein A-I gene (ApoA1)^[34]^. Taken together, these studies suggest that induction of Egr1 in response to insulin is vital to insulin’s action on liver metabolism.

### Egr1, insulin resistance, and obesity

Insulin resistance is a central defect in type 2 diabetes mellitus (T2DM). The link between Egr1 and insulin resistance is originally from the observation that Egr1 mRNA is highly increased in adipocytes from diabetic mice^[35]^. PI3K/Akt pathway is activated upon insulin stimulation, which is required for glucose uptake and glycogenesis to lower circulating glucose level^[36]^. Meanwhile, insulin stimulates the activation of MAPK (ERK1 and 2) that promotes insulin resistance^[37]^. Thus, the balance between PI3K/Akt and MAPK signaling pathway is critical to maintain insulin sensitivity. Egr1 transcriptionally regulates phosphatase and tensin homologue (PTEN), a suppressor of PI3K/Akt signaling^[38]^. Meanwhile, Egr1 regulates geranylgeranyl pyrophosphate synthase (GGPPS), an activator of ERK/MAPK signaling^[39]^. Thus, inhibiting Egr1 in adipocyte simultaneously blocks MAPK signaling and augments PI3K/Akt signaling, and subsequently improves insulin sensitivity^[40]^. Collectively, these studies suggest that pharmacological targeting adipocyte Egr1 could be potentially applied for developing novel treatment for T2DM.

Obesity commonly coexists with Insulin resistance. The link of Egr1 to obesity and obesity-associated fatty liver has been reported in mouse studies. For example, whole body *Egr1*-deficient mice fed a high fat diet are less susceptible to diet-induced obesity and obesity-associated disorders such as insulin resistance, hyperinsulinemia, hyperlipidemia, and fatty liver, which largely depends on the increase of energy expenditure in the adipose tissue of *Egr1*-null mice^[20]^. These studies suggest that the upregulation of Egr1 in adipocytes is involved in promoting metabolic disorders and that targeting Egr1 in adipocyte could be useful for the obesity treatment.

The report of Egr1 function in liver steatosis is somehow contradictory. One earlier study showed that Egr1 expression levels in the liver are positively correlated to high caloric intake in mice, humans, and non-human primates^[41]^. In addition, whole-body *Egr1*^−/−^ mice are protected from chronic ethanol-induced fatty liver due to the decreased expression and release of TNFα from macrophages^[42]^. However, recent studies highlight that increasing Egr1 levels in the liver ameliorates diet-induced fatty liver disease. For example, the white pitaya (hylocereusundatus) juice attenuates diet-induced liver steatosis and improves insulin sensitivity in C57BL/6J mice, which is accompanied by an increase in hepatic Egr1 mRNA level^[43]^. Thus, future research focusing on hepatocyte-specific Egr1 function in liver metabolism will be very valuable.

### Egr1 and cholesterol biosynthesis

Cholesterol is an essential component for cell membrane and serves as the precursor to all steroid hormones. However, high intracellular cholesterol is toxic to cells and high blood levels of cholesterol increase the risk for atherosclerosis development^[44]^. Therefore, the overall cholesterol level is tightly controlled in the body. The liver plays a central role in this regulation by balancing multiple pathways involved in *de novo* cholesterol biosynthesis, cholesterol conversion to bile acids, biliary cholesterol excretion, and reverse cholesterol transport^[45]^. Sterol response element binding proteins (SREBPs) are important transcription factors that regulate expression of genes in lipid metabolism including fatty acids and cholesterol synthesis. Three isoforms (SREBP-1a, SREBP-1c, and SREBP-2) have been identified in mammals. SREBP-1 mainly regulates genes required for fatty acid biosynthesis and SREBP-2 is responsible for the induction of genes involved in cholesterol biosynthesis and uptake, including HMG-CoA synthase (*Hmgcs*) and low-density lipoprotein receptor (*Ldlr*)^[46]^.

Egr1 regulates the expression of cholesterol biosynthetic genes, such as *Hmgcs*, farnesyl-diphosphate synthase (*Fdps*), farnesyl-diphosphate farnesyltransferase 1 (*Fdft1*), lanosterol synthase (*Lss*), sterol-4α-carboxylate 3-dehydrogenase (*Nsdhl*), and malic enzyme (*Me1*), in rat hepatomaH4IIE cells^[24]^. Additionally, Egr1 acts in concert with SREBP-2 to mediate insulin-induced cholesterol biosynthesis in the liver^[24]^. Oncostatin M (OM) is a gp130 family member produced by the F4/80-positive macrophages^[47]^. In human hepatoblastoma HepG2 cells, Egr1 is induced by OM and binds to the sterol-independent regulatory element (SIRE) in LDLR promoter region with co-activator CCAAT/enhancer binding protein-beta (C/EBPβ) and activates LDLR transcription^[48,49]^. Together, these studies point to Egr1 as an important modulator of cholesterol metabolism in the liver.

## EGR1 AND LIVER REGENERATION

The liver has a tremendous capacity to regenerate after injury, which is a highly coordinated process involving both liver parenchymal and non-parenchymal cells. During liver regeneration, adult hepatocytes enter the cell cycle (G0 to G1) and progress through the cell cycle (G1 to M) until liver mass is restored^[50]^. Many signals regulate the process of liver regeneration^[51]^. For example, lipopolysaccharide and cytokines are important mediators of the initiation phase^[52]^. Growth factors such as hepatocyte growth factor (HGF) and epidermal growth factor (EGF) regulate the progression phase^[53]^. TGF-β1 signals later terminate hepatocyte proliferation^[54]^. Additionally, growth arrest-specific 1 (Gas1), a cell proliferation inhibitor, is induced during liver regeneration at the cycle G1/S transition, contributing to the final termination of regeneration^[55]^. Perturbations in the liver-regenerative response cause prolonged liver injury and delayed liver recovery.

The role of Egr1 in liver regeneration was first suggested by animal studies demonstrating that Egr1 was immediately induced during the initiation phase of liver regeneration^[56,57]^. Using a transgenic Egr1 luciferase (Egr1-luc) mouse model, Dussmann *et al*.^[14]^ demonstrated that Egr1 expression was increased at the site of wound healing in partial hepatectomy. Another earlier study showed that Egr1 expression significantly increased after 15 min and subsided within 60 min after partial hepatectomy in rat livers^[56]^. More recent studies in mice have extended the peak of Egr1 induction to 12 h in partial hepatectomy-induced liver regeneration^[58]^ and to 2 h in carbon tetrachloride (CCl_4_) exposure-induced liver regeneration^[18]^. The specific signals that regulate Egr1 expression during liver regeneration are not quite understood, a number of candidates are worthy of consideration. For example, extracellular ATP has been implicated as a potent stimulus for Egr1 expression^[59]^. P2Y purinoceptor 2 (P2Y2) is a G protein coupled receptor that is activated by ATP in hepatocytes. The fact that the induction of Egr1 is impaired in *P2Y2*^−/−^ liver subjected to partial hepatectomy indicates that P2Y2 may regulate Egr1 expression during liver regeneration^[60]^. Additional candidates that regulate Egr1 expression are likely to include interleukin-6 (IL-6) and C/EBPβ, because the induction of Egr1 has been shown to be impaired in *IL-6*^−/−^ or *C/EBPβ*^−/−^ liver subjected to partial hepatectomy^[61,62]^.

EGR1 is essential for cell-cycle entry and progression during liver regeneration as Egr1 directly regulates cell cycle mediators. Lai *et al.*^[63]^ found that *Egr1*-deficient mouse livers had a substantially lower recovery rate after liver injury, which was accompanied with the reduced expression of cell cycle mediators such as Cyclin D1, Cyclin E, and proliferating cell nuclear antigen. After subcutaneous administration of CCl_4_, *Egr1*-deficient mice exhibited increased liver injury and delayed cell cycle progression^[18,58]^. Acute ethanol dosing of *Egr1*^−/−^ mice also resulted in exacerbated liver injury associated with impaired liver repair^[64]^. Collectively, these studies suggest that Egr1 and its regulated cell-cycle entry and progression is critical for liver regeneration. Additionally, Egr1 contributes to the regulation of a large number of genes required for the regenerative response, including cell division cycle 20 (cdc20), a key regulator of the mitotic anaphase-promoting complex, and cytokines necrosis factor-alpha (TNFα), IL-6, and lymphotoxin-beta^[14,18,57,65,66]^. Therefore, Egr1 plays a critical role in liver regeneration after injury.

## EGR1 IN LIVER FIBROSIS AND ACETAMINOPHEN-INDUCED HEPATOTOXICITY

Liver fibrosis is the wound-healing response of the liver to chronic injury that entails cell proliferation, inflammation, angiogenesis, as well as synthesis and remodeling of extracellular matrix^[67–70]^. Prolonged tissue injury can lead to excessive accumulation of extracellular matrix in the organ, a hallmark of fibrosis. Egr1 has been shown to induce transcription of growth factors and stimulate collagen production in human fibroblasts and fibrosarcoma cells, suggesting the contribution of Egr1 to fibrogenesis ^[22,71]^. TGF-β1, a key regulator of fibrogenesis, is an upstream regulator of Egr1^[10]^; however, Egr1 also regulates the expression of TGF-β1 in response to the hepatitis B virus^[72]^, which hints to the existence of a possible feedback regulation between TGF-β1 and Egr1 during fibrogenesis.

Acetaminophen (APAP) is widely used to treat pain and reduce fever. APAP is mainly metabolized by the liver, undergoing glucuronidation, sulfation, or N-hydroxylation. The sulfate product is the primary, non-toxic metabolite in children; whereas, the glucuronide metabolite is the primary, non-toxic metabolite in adults. The hydroxylated product is the bioactivation of APAP by cytochrome 2E1 (Cyp2E1) that leads to the toxic, reactive metabolite, N-acetyl-p-benzoquinone imine (NAPQI). The final attempt to prevent toxicity is to conjugate NAPQI to glutathione^[73]^. In the event of APAP overdose, the glutathione stores are depleted; the reactive metabolite binds to hepatic proteins, leading to hepatic necrosis. In western countries, acute liver injury due to APAP overdose is the main cause for drug-induced acute liver failure^[74]^. In addition, long-term application of APAP has been linked to the increased hepatic inflammation and liver fibrosis in patients^[75]^.

The report of Egr1 function in acute or chronic APAP-induced hepatotoxicity is contradictory. In an acute APAP-induced liver injury mouse model, both Egr1 mRNA level and transcriptional activity in hepatocytes are increased^[76]^. Inhibition on ERK1/2-mediated Egr1 transcriptional activation by caffeic acid (an organic compound found in coffee, fruit, and herbs) attenuates APAP-induced hepatotoxicity^[76]^, suggesting that inhibiting Egr1 activation is beneficial to protect against APAP-overdose induced acute hepatotoxicity. By contrast, a recent study using WT and *Egr1*^−/−^ mice in chronic APAP-induced liver injury has demonstrated that *Egr1*^−/−^ livers exhibited a more severe hepatotoxicity and fibrotic response compared to WT mice under APAP overdose^[77]^. Collectively, these data support Egr1 as an important mediator in APAP-induced hepatotoxicity and liver fibrosis; however, whether Egr1 could act as an inducer or protector against APAP-induced liver injury has remained elusive. Additional studies using cell-type specific *Egr1*-deficient animals to determine the involvement of Egr1 in acute and chronic APAP-induced liver injury would be highly beneficial for a more clear definition of cell-type specific role of Egr1 in liver injury and fibrosis.

## EGR1 AND LIVER CANCER

Egr1 is demonstrated to act as both a tumor suppressor and a tumor promoter in cancers. The tumorigenic role of Egr1 was described in prostate, skin and kidney cancers^[78]^. By contrast, tumor suppressor activity of Egr1 was reported in fibrosarcoma, glioblastoma, lung and breast cancers^[79,80]^. The role of Egr1 in liver cancers remains elusive, as studies evaluating the role of Egr1 in liver cancer development and progression have reported contradicting conclusions.

Accumulating studies suggest Egr1 as a tumor suppressor in HCC. Egr1 is commonly downregulated in HCC tissues from humans and murine, indicating that the downregulation of Egr1 is related to HCC development^[81]^. However, mechanisms responsible for the downregulation of Egr1 in liver cancer remain unknown. A recent study has described that EGR1 carries mutational intratumoral heterogeneity and frameshift mutations in colorectal and gastric cancers which have high microsatellite instability^[82]^. Thus, it could be interesting to know whether the same mechanism could exist in liver cancer and contribute to the decrease of EGR1. Aberrant MAPK signaling activation is a key player in driving tumor proliferation^[83–85]^. Inhibition of P42/44MAPK in HepG2 cells leads to suppression on cell growth, proliferation, and survival, accompanied by an induction of Egr1 in tumor cells^[86]^. Recently, (125)I-UdR radionuclide therapy combined with Egr1-promoter-based interferon gamma (*IFNγ*) gene therapy was described to efficiently reduce tumor proliferation and promote animal survivals in mice bearing H22 hepatomas^[87]^. Overexpression of Egr1 decreases the growth rate and tumorigenicity of the HCC cell line HHCC cells^[88]^. Furthermore, Egr1 induces apoptosis in human hepatoma cells (HepG2 and Hep3B) that can be enhanced by synthetic chenodeoxycholic acid derivative, HS-1200^[89]^. Collectively, these studies have demonstrated that Egr1 functions as a tumor suppressor in HCC via inhibiting tumor proliferation and promoting apoptosis.

In addition, Egr1 regulates the expression of a large number of genes required for suppressing HCC growth, including PTEN^[38]^, a very well known tumor suppressor that inhibits PI3K signaling pathway in HCC. EGR1 protein sumoylation is required for activation of PTEN transcription, in which the phosphorylation of EGR1 by AKT at S350 and T309 allows EGR1 protein sumoylation^[90]^. In addition, Egr1/PTEN axis is essential for ribonucleotide reductase regulatory TP53 inducible subunit M2B (RRM2B) inhibition on HCC cell migration^[91]^. Recently, Wang *et al.*^[92]^ has described a cascade, involving Egr1, microRNA-203a (miR-203a), and homeobox D3 (HOXD3), inhibits HCC tumorigenesis. Through both *in vitro* and *in vivo* studies, the authors have demonstrated that Egr1 directly activates miR-203a expression by binding to the miR-203a promoter that results in suppression on HOXD3^[92]^. Taken together, these studies support an anti-tumor role of Egr1 in HCC.

Contrasting the anti-tumorigenic role of Egr1 is study indicating that Egr1 is associated with HCC tumorigenesis. In a study using cDNA microarray and chromatin immunoprecipitation (ChIP) assay to assess the genes associated with tumor angiogenesis, Egr1 is identified as a key player to mediate HGF-induced upregulation of vascular endothelial growth factor and IL-8^[93]^. In an attempt to identify early biomarkers of HCC, Archer *et al.*^[94]^ has performed gene expression microarray analyses in HCC tissues and revealed that Egr1 and vesicle associated membrane protein-2 are positively correlated to hepatitis virus-induced HCC. Additionally, G protein-coupled receptor kinase2 overexpression reduces insulin-like growth factor 1-induced HCC cell proliferation and migration that is mediated by decreasing Egr1^[95]^. All these studies suggest that activation of Egr1 might promote HCC development.

Additionally, Egr1 is described to contribute to hypoxia-induced HCC cells’ resistance against anticancer drugs^[74,96]^. One of the proposed mechanisms behind such phenomenon connects Egr1, hypoxia, and microtubules. Egr1 is co-localized with microtubules and mediates hypoxia-induced stabilization of microtubules from disassembly^[96]^. Expected, knockdown of Egr1 improves drug effectiveness under hypoxic conditions^[96]^. Another mechanism connects Egr1, hypoxia, and autophagy to HCC drug resistance. Autophagy contributes to the HCC cells resistance against chemotherapeutic agents under hypoxic conditions^[97–99]^. Egr1 transcriptionally regulates hypoxia-induced autophagy by binding to the promoter of microtubule-associated protein 1 light chain 3 and promotes autophagosomes formation in HCC cells^[74]^. Collectively, these studies suggest that inhibiting Egr1 expression or function to increase tumor cells’ sensitivity to chemotherapeutics could be applied as a novel approach for HCC therapy. In addition, whether the current discrepancies on Egr1 function in HCC could be due to a dual role of Egr1 during HCC development, first acting as an activator and then as a repressor, still remains elusive and requires further investigation.

## CONCLUSION

**Figure 2:**
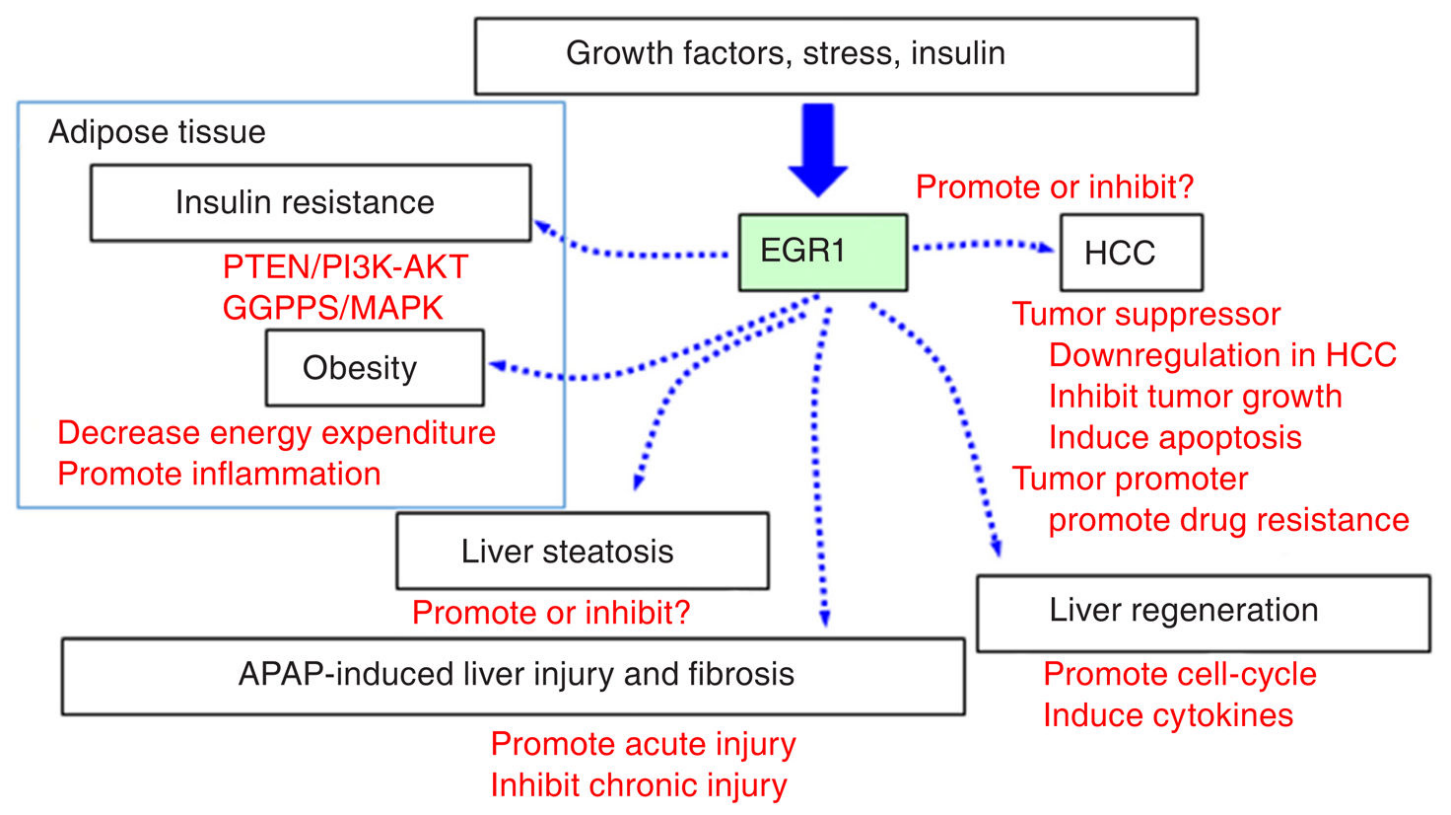
Model of EGR1 function in metabolic diseases and liver diseases. EGR1 is induced in response to various stimuli such as growth factors, stress, and insulin signal. EGR1 regulates a wide array of transcriptional targets involved in multiple biological functions related to lipid and glucose metabolism. In particular, increase of EGR1 in adipose tissue is associated with insulin resistance and obesity. In the liver, dysregulation of EGR1 is associated with liver steatosis. EGR1 promotes acute acetaminophen (APAP)-induced liver injury while attenuates chronic APAP-induced liver fibrosis. EGR1 is important for liver regeneration as it promotes cell-cycle entry and progression, as well as stimulates production of cytokines required for tissue repair. Finally, dysregulation of EGR1 associates with HCC development. EGR1 regulates HCC tumor growth and apoptosis, and is involved in hypoxia-induced drug resistance. EGR1: early growth response 1; HCC: hepatocellular carcinoma

As a Zinc-finger transcription factor, Egr1 has a diverse range of functions implicated in various cell types. The major roles of Egr1 in liver diseases are summarized and depicted in Figure 2. Research efforts using various animal models such as fatty liver, liver injury and fibrosis have contributed greatly to the elucidation of Egr1 liver-specific function. However, in some instances, such as in insulin signaling as well as HCC studies, the data regarding the role of Egr1 are contradictory. Hence, much progress is required to uncover and characterize the role of Egr1 in various types of cells in the regulation of normal liver function. For example, studying the effects of insulin signaling, APAP, ethanol, or CCL_4_ in hepatocyte-specific or macrophage-specific *Egr1* knockout models are greatly appreciated. Utilization of primary cell cultures (such as hepatocytes, stellate cells, and macrophages) from normal liver to assess Egr1 functions may also aid in elucidation of liver-specific Egr1 regulation. On the other hand, due to its regulation of key fibrotic mediators, Egr1 may be a promising target for anti-fibrotic therapy. Overall, much progress is required to uncover and characterize the cell-type specific role of Egr1 in the liver. Improving our understanding of Egr1 in liver metabolism and liver cancer may provide new insights to facilitate developing novel treatments or prevention strategies for liver diseases.
